# Lower Low-Density Lipoprotein Cholesterol Levels Are Associated with Severe Dengue Outcome

**DOI:** 10.1371/journal.pntd.0003904

**Published:** 2015-09-03

**Authors:** Hope H. Biswas, Aubree Gordon, Andrea Nuñez, Maria Angeles Perez, Angel Balmaseda, Eva Harris

**Affiliations:** 1 Division of Epidemiology, School of Public Health, University of California, Berkeley, Berkeley, California, United States of America; 2 Department of Epidemiology, School of Public Health, University of Michigan, Ann Arbor, Michigan, United States of America; 3 Laboratorio Nacional de Virología, Centro Nacional de Diagnóstico y Referencia, Ministry of Health, Managua, Nicaragua; 4 Hospital Infantil Manuel de Jesús Rivera, Managua, Nicaragua; 5 Division of Infectious Diseases and Vaccinology, School of Public Health, University of California, Berkeley, Berkeley, California, United States of America; Pediatric Dengue Vaccine Initiative, UNITED STATES

## Abstract

Dengue virus (DENV) is a flavivirus of worldwide importance, with approximately 4 billion people across 128 countries at risk of infection, and up to 390 million infections and 96 million clinically apparent cases estimated annually. Previous *in vitro* studies have shown that lipids and lipoproteins play a role in modifying virus infectivity. However, the relationship between development of severe dengue and total cholesterol, high-density lipoprotein cholesterol (HDL-C), and low-density lipoprotein cholesterol (LDL-C), respectively, is unclear. We analyzed data from 789 laboratory-confirmed dengue cases and 447 other febrile illnesses (OFI) in a prospective pediatric hospital-based study in Managua, Nicaragua between August 2005 and January 2013, using three different classifications of dengue severity: World Health Organization (WHO) 1997, WHO 2009, and standardized intervention categories. Total serum cholesterol and LDL-C levels decreased over the course of illness and were generally lower with increasing dengue severity, regardless of classification scheme. Greater decreases in LDL-C than HDL-C were observed among dengue-positive patients compared to patients with OFI and among severe dengue compared to mild dengue cases. Furthermore, daily cholesterol levels declined with daily albumin blood levels. To examine the effect of cholesterol at presentation on subsequent risk of development of severe dengue, relative risks and 95% confidence intervals were calculated using multivariable modified Poisson models. We found that lower total serum cholesterol and LDL-C levels at presentation were associated with subsequent risk of developing dengue hemorrhagic fever/dengue shock syndrome using the WHO 1997 dengue severity classification, and thus that the reduction in LDL-C is likely driving the decreases observed in total serum cholesterol levels among dengue-positive patients. Our results suggest that cholesterol blood levels are important correlates of dengue pathophysiology and should be explored as part of a prognostic biomarker panel for severe dengue.

## Author Summary

Dengue is a viral infection of worldwide importance with up to 96 million cases annually. Cholesterol, a type of lipid, may play a role in dengue virus infectivity and severity, but changes in cholesterol levels over the course of illness are not well-understood. To investigate the relationship between development of severe dengue and total cholesterol, high-density lipoprotein cholesterol (HDL-C), and low-density lipoprotein cholesterol (LDL-C), we analyzed data from a prospective hospital-based study of pediatric dengue in Managua, Nicaragua (August 2005–January 2013). Because different classifications of dengue severity are used, we performed analyses using three different classifications. Cholesterol decreased over the course of illness and differed across disease outcome, regardless of classification scheme. Greater decreases in LDL-C than HDL-C were observed among dengue-positive patients versus patients with other febrile illnesses and among severe versus mild dengue cases. In multivariate models, lower total serum cholesterol and LDL-C levels at presentation were associated with subsequent risk of developing dengue hemorrhagic fever/dengue shock syndrome (World Health Organization 1997 classification). Our results suggest that reduction in LDL-C is likely driving decreases observed in total serum cholesterol levels among dengue-positive patients and should be explored as part of a prognostic biomarker panel for severe dengue.

## Introduction

Dengue virus (DENV) is a flavivirus of worldwide importance, with approximately 4 billion people across 128 countries at risk of DENV infection [1]. Of the estimated 390 million annual DENV infections, 96 million are symptomatic, and a subset of individuals develop severe forms of the disease, which consist of hemorrhagic manifestations and vascular leakage, leading to hypovolemic shock [2,3]. *In vitro* studies of the pathophysiology of DENV and other flavivirus (e.g., Japanese encephalitis virus, West Nile virus) infections suggest that lipids and lipoproteins may play a role in modifying virus infectivity of target cells. Cholesterol-rich lipid rafts have been shown to be required for flavivirus entry [4–6], and the related hepatitis C virus enters host cells via low-density lipoprotein (LDL) receptors [7]. The addition of cholesterol during viral adsorption blocks Japanese encephalitis virus and DENV infectivity [4]. Further, lovastatin, an inhibitor of cholesterol synthesis, also inhibits DENV replication [8,9] and is currently in clinical trials as a potential dengue therapeutic [10]. After infection, DENV, West Nile virus and Japanese encephalitis virus mimic or hijack lipid metabolic pathways [9,11–15] by increasing lipid raft formation, intracellular levels of total cholesterol, and LDL receptors on the surface of infected cells [15]. Together, these studies suggest that cholesterol is beneficial for DENV replication and that DENV infection modulates cholesterol metabolism.

Previous clinical studies have generally shown lower levels of plasma and serum cholesterol among severe dengue (dengue hemorrhagic fever/dengue shock syndrome [DHF/DSS]) cases compared to less severe dengue cases or healthy controls [16–20], possibly driven by a reduction in LDL cholesterol (LDL-C) [20]. Total cholesterol is comprised of LDL-C, high-density lipoprotein cholesterol (HDL-C), and triglycerides. However, the relationship between severe dengue and total cholesterol, HDL-C, and LDL-C, respectively, is unclear. In the two studies that used multivariable models to examine the relationship between cholesterol and severe dengue, HDL-C and LDL-C were associated with severe dengue outcome in one of these studies [18], but not the other [19]. Total serum cholesterol was not associated with severe dengue outcome [19] or was not separately analyzed [18]. However, neither of these studies fully accounted for the time ordering of cholesterol level in relation to development of severe dengue outcome. Without time ordering, it is impossible to determine whether cholesterol level affects development of severe dengue or is a result of developing severe dengue.

In this study, we sought to delineate the trajectories of cholesterol levels over time by DENV infection status in order to understand the effect of the response to DENV infection on cholesterol levels. We also sought to delineate their trajectories by dengue severity to understand how cholesterol levels change over time among patients who develop severe dengue. Lastly, we aimed to assess the association of cholesterol level at presentation with development of severe dengue. To address these questions, we analyzed data from a prospective hospital-based study of pediatric dengue cases in Managua, Nicaragua, between August 2005 and January 2013. Because different classifications of dengue severity are used in the literature, we performed analyses using three different classifications of severity: the World Health Organization (WHO) 1997 classification criteria [21], the WHO 2009 classification criteria [22] and standardized intervention categories [23,24]

## Methods

### Study site and population

A prospective study was conducted from 2005 to the present in the Infectious Disease Ward of the Hospital Infantil Manuel de Jesús Rivera in Managua, Nicaragua, to study clinical, immunological and viral risk factors for severe dengue. This hospital is the National Pediatric Reference Hospital and treats the vast majority of children seeking tertiary care in Managua and referred from around the country [25]. Infants and children between six months and 14 years of age with fever or history of fever <7 days and one or more of the following signs and symptoms: headache, arthralgias, myalgias, retro-orbital pain, positive tourniquet test, petechiae, or signs of bleeding were eligible to participate in the study. Patients with a defined focus of infection other than dengue or who were actively enrolled in the concurrent Pediatric Dengue Cohort Study [26] were excluded. Children weighing <8 kg, children <6 months of age, and children ≥6 years of age displaying signs of altered consciousness at the time of recruitment were also excluded. For the current analysis, we also excluded children <1 year of age, due to the possible presence of maternal antibodies, as well as children with nephrotic syndrome or obesity (body mass index (BMI) ≥32), due to abnormally high cholesterol levels. Both inpatients and outpatients were enrolled each year during the peak of the dengue season (August 1 to January 31) and followed clinically through the acute phase of illness.

Upon enrollment, a medical history was taken and a complete physical exam was performed. Clinical data, including vital signs, signs and symptoms, and fluid balance and treatment, were recorded twice daily on standardized data collection forms during hospitalization or through daily ambulatory visits by the same team of study physicians and nurses responsible for care of hospitalized study participants. A blood sample was also collected daily for three days for complete blood counts with platelets, blood chemistry, and serological, virological and molecular biological tests for DENV infection. Additional samples for platelet count and hematocrit measurements were collected as necessary. A convalescent serum sample (14–21 days post-onset of illness) was also collected for paired serological testing. Participants were hospitalized if they presented any of the following warning signs: abdominal pain or tenderness; persistent vomiting; clinical fluid accumulation; mucosal bleeding; lethargy/restlessness; liver enlargement; or increase in hematocrit concurrent with rapid decrease in platelet count.

### Ethics and STROBE statement

The protocol for this study was reviewed and approved by the Institutional Review Boards of the University of California, Berkeley, and the Nicaraguan Ministry of Health. Parents or legal guardians of all participants provided written informed consent, and participants 6 years of age and older provided assent. This study conforms with the STROBE reporting guidelines (see S4 Table).

### Data collection

All information was collected every 12 hours for inpatients and every 24 hours for outpatients who were asked to return on a daily basis on Case Report Forms (CRFs) designed to follow patients’ progress, with vital signs and fluid intake/output recorded more often as appropriate. Each CRF was completed by an infectious disease pediatrician and reviewed independently by a second physician. Following this review, the CRF information was entered into an Access 2003 database by double-data entry and was systematically monitored by weekly quality control checks.

### Cholesterol measurements

For inpatients, a non-fasting blood sample was obtained each morning to measure serum lipids. For outpatients, a non-fasting blood sample was obtained at each follow-up visit. Total serum cholesterol, HDL-C (direct) and LDL-C (direct) were measured using the CHOD-PAP method (CHOD: cholesterol oxidase; PAP: phenol plus aminophenazone). Total serum cholesterol and HDL-C were measured throughout the study; LDL-C was measured from August 2007 until the present. From August 2005 to July 2007, the BioCon kit was used for cholesterol measurements, and reactions were read in a spectrophotometer. From August 2007 to the present, cholesterol was measured using the same CHOD-PAP method, but using the Cobas Integra 400 platform and the corresponding cholesterol kit (Roche Diagnostics). The National Reference Laboratory of the Nicaraguan Ministry of Health performed all assays throughout the study. Two quality control systems were used. For the first, the kit’s internal controls and calibration curve were processed for each run. For the second, cholesterol measurements were processed together with two control sera with normal and pathological values for each run (Precinorm U and Precipath U, Roche Diagnostics, respectively). The machine automatically checks for the values of the two control sera and validates the run if these values are within the expected range. Moreover, the laboratory participates every 6 months in an external quality assessment program run by the United Kingdom National External Quality Assessment Service, Clinical Chemistry (Birmingham), a WHO Collaborating Centre. In each assessment, blinded samples are sent by UK NEQAS for processing in the laboratory and results on 25 parameters, including cholesterol, are sent back for review. To date, all reviews have been successful.

### Dengue diagnosis

A case was considered laboratory-confirmed dengue when acute DENV infection was demonstrated by: detection of DENV RNA by RT-PCR; isolation of DENV; seroconversion of DENV-specific IgM antibodies observed by MAC-ELISA in paired acute- and convalescent-phase samples; and/or a ≥4-fold increase in anti-DENV antibody titer measured using Inhibition ELISA in paired acute- and convalescent-phase samples [27–30]. DENV serotypes were identified by RT-PCR and/or virus isolation [31,32]. Patients who tested negative for DENV infection were considered patients with other febrile illness (OFI).

### Dengue disease outcome

Laboratory-confirmed dengue cases were classified by severity using a computerized algorithm that compiled all clinical data meeting each criterion for each of the disease classifications as detailed in the WHO Guidelines (S1 Table). Dengue Fever (DF), DHF and DSS were defined according to the 1997 WHO classification criteria (S2 Table) [21]. Laboratory-confirmed dengue cases were also classified according to the 2009 revised WHO classification criteria (Dengue with and without Warning Signs, Severe Dengue; S2 Table) [22] and the three standardized clinical intervention levels that were established in the DENCO study sponsored by the WHO Special Programme for Research and Training in Tropical Diseases (Categories 1, 2, and 3; S2 Table) [23,24]. The time-point at which the outcome occurred was defined as the day on which the patient’s cumulative signs and symptoms met the WHO or standardized intervention criteria. Dengue cases were defined as primary DENV infections if the convalescent antibody titer was <2,560, and as secondary infections if the convalescent antibody titer was ≥2,560, as determined by Inhibition ELISA [33]. A case was considered indeterminate if RT-PCR yielded negative results, no DENV was isolated, and a convalescent sample could not be obtained.

### Statistical analysis

Data from August 1, 2005, through January 31, 2013, were used for analysis. To delineate the trajectories of cholesterol by DENV infection status, we used repeated measures linear regression (an exchangeable, working-within-subject correlation model via a generalized estimating equation [34]) to estimate population average rates of change in levels of total serum cholesterol, LDL-C and HDL-C. Time-varying cholesterol was treated as the outcome and modeled by age, gender, DENV infection status, day of illness and an interaction term for DENV infection status and day of illness in the regression. The day of fever onset was defined as day 1 of illness. Only data from days 2 to 8 of illness were included in the analysis because the counts before and after this period did not allow for meaningful comparisons. It was necessary to use a statistical method that accounts for repeated measures because cholesterol levels measured on the same subjects over time are expected to be correlated. The generalized estimating equation approach corrects for within-subject correlations over time and provides robust standard error estimates that are resistant to model misspecification [35].

After fitting the regression models, we predicted the marginal mean cholesterol levels for each day of illness separately by DENV infection status, weighted by the distribution of age and gender in the study population using a marginal standardization approach. Marginal standardization is a method that estimates predicted probabilities after models are adjusted for confounders, allowing inference to the overall population from which the study data were drawn [36]. The 95% confidence intervals (CIs) were calculated for the marginal means using the delta method, and a global test for multiple comparisons was performed across all time-points using the margins command with the contrast option in STATA 13/SE (StataCorp LP, College Station, TX) [37]. This global test assesses whether there is an overall difference between groups and was used instead of Bonferroni, which would have likely been an overcorrection [38]. The marginal means and their 95% CIs were then plotted by DENV infection status and day of illness.

We repeated this analysis to delineate the trajectories of cholesterol by dengue severity. For each dengue severity classification, time-varying cholesterol was treated as the outcome and modeled by age, gender, severe dengue outcome, day of illness and an interaction term for severe dengue outcome and day of illness in the regression. We restricted the analysis to patients who were classified as mild dengue at presentation and, if they developed severe dengue, developed severe dengue >12 hours after presentation. For the WHO 1997 classification, mild dengue was defined as dengue fever (DF) and severe dengue was defined as dengue hemorrhagic fever or dengue shock syndrome (DHF/DSS). For the WHO 2009 classification, mild dengue was defined as dengue with or without warning signs (DWS) and severe dengue was defined verbatim (SD). For the standardized intervention categories, mild dengue was defined as intervention category (IC) 1/IC 2 care and severe dengue was defined as IC 3 care.

To examine the effect of cholesterol level at presentation on subsequent risk of development of severe dengue, relative risks (RRs) and 95% CIs were calculated using modified Poisson models with robust standard errors. The modified Poisson approach uses a robust error variance procedure to provide a consistent and efficient estimate of the relative risk [39]. We again restricted the analysis to patients who were classified as mild dengue at presentation and, if they developed severe dengue, developed severe dengue >12 hours after presentation (n = 108 using the WHO 1997 classification, n = 101 using the WHO 2009 classification, and n = 164 using standardized intervention categories) to ensure that we had appropriate time ordering of the exposure (cholesterol) before the outcome (severe dengue). We constructed a directed acyclic graph [40] to characterize the pathways through which cholesterol at presentation could be associated with development of severe dengue (see S1 Fig) and adjusted models for the following confounders based on this working diagram: age (years), gender, immune response (secondary vs. primary) and malnutrition. For children ≥2 years of age, malnutrition was defined as less than the third BMI-for-age percentile according to growth charts by the Centers for Disease Control and Prevention [41]; for children <2 years of age, malnutrition was defined as a deficit of ≥10% of the ideal weight based on the Gómez classification [42]. We repeated this analysis for each dengue severity classification.

Lastly, we examined the association of cholesterol levels with albumin levels (as a measure of vascular permeability) using repeated measures linear regression, adjusting for DENV infection status and day of illness. Median total serum cholesterol levels were then plotted against median albumin levels by DENV infection status and day of illness. All analyses were performed using STATA 13/SE.

## Results

**Fig 1:**
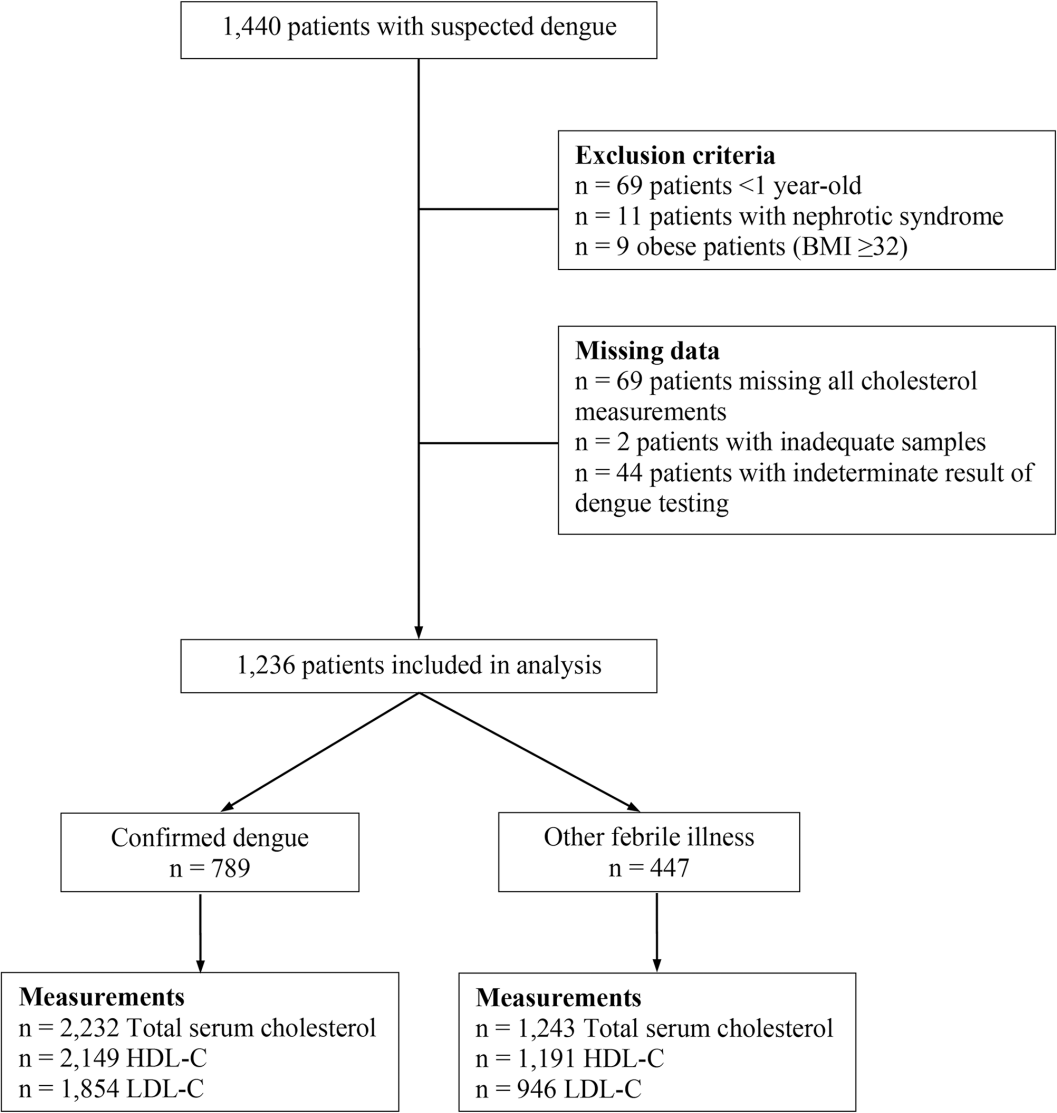
Eligibility flow chart. Of the 1,440 patients who presented to the hospital with suspected dengue, 1,236 met the eligibility criteria and were included in the analysis. Abbreviations: BMI, body mass index.

Of the 1,440 patients in the dengue hospital study, 69 patients <1 year of age, 11 patients with nephrotic syndrome, and 9 patients with obesity (BMI ≥32) were excluded from the analysis (see Fig 1). An additional 69 patients missing all cholesterol measurements, 2 patients with inadequate samples for laboratory testing, and 44 patients with an indeterminate result of dengue testing were excluded, leaving 1,236 patients available for analysis. Of the 1,236 patients, 789 (64%) were laboratory-confirmed as DENV-positive. Among the dengue cases, 149 were classified as DHF and 48 as DSS using the 1997 WHO classification, and 66 were classified as Dengue without Warning Signs, 466 as Dengue with Warning Signs, and 257 as Severe Dengue using the 2009 WHO classification. Of the 257 with Severe Dengue, 94 had hypotensive shock, 180 had compensated shock, 74 had fluid accumulation with respiratory difficulty, 2 had alteration in AST or ALT, 9 had change in CNS, and 52 had suspected myocardiopathy. The remaining 447 patients (36%) tested negative for DENV and were classified as OFI.

The characteristics of the 1,236 study participants included in the analysis are summarized in Table 1. Overall, similar proportions of males and females were classified as different disease severity categories in both WHO 1997 and 2009 classification schemes and the IC classification scheme. Compared to other age groups, children aged 9 to 12 years were more likely to be classified as DHF, DSS, SD, and IC 3. Dengue cases were more likely to have a secondary immune response, and approximately half were DENV-3 serotype. Most patients (82%) were hospitalized. Patients generally presented on day 4 or 5 of illness and were hospitalized for a median of 3 days. Patient counts by day of illness and WHO classification are shown in S3 Table. Median cholesterol levels by day of illness and disease severity classification are shown in S2 Fig.

**Table 1:** Characteristics of study participants by DENV infection status and disease severity classification criteria.

					WHO 1997		WHO 2009		IC	
		Total	DENV+	OFI	DF	DHF/DSS	DWS	SD	1/2	3
		(n = 1,236)	(n = 789)	(n = 447)	(n = 592)	(n = 197)	(n = 532)	(n = 257)	(n = 588)	(n = 201)
Characteristic		n (%)	n (%)	n (%)	n (%)	n (%)	n (%)	n (%)	n (%)	n (%)
**Sex**	Female	604 (49)	397 (50)	207 (46)	298 (50)	99 (50)	271 (51)	126 (49)	298 (51)	99 (49)
	Male	632 (51)	392 (50)	240 (54)	294 (50)	98 (50)	261 (49)	131 (51)	290 (49)	102 (51)
**Age (years)**	1–4	285 (23)	146 (18)	139 (31)	112 (19)	34 (17)	103 (19)	43 (17)	115 (20)	31 (15)
	5–8	385 (31)	250 (32)	135 (30)	199 (34)	51 (26)	183 (34)	67 (26)	195 (33)	55 (27)
	9–12	399 (32)	276 (35)	123 (28)	201 (34)	75 (38)	173 (33)	103 (40)	190 (32)	486 (43)
	≥13	167 (14)	117 (15)	50 (11)	80 (13)	37 (19)	73 (14)	44 (17)	88 (15)	29 (15)
**Immune response**	Primary	N/A	346 (44)	N/A	302 (51)	44 (22)	255 (48)	91 (35)	276 (47)	70 (35)
	Secondary	N/A	414 (52)	N/A	267 (45)	147 (75)	260 (49)	154 (60)	295 (50)	119 (59)
	Indeterminate	N/A	29 (4)	N/A	23 (4)	6 (3)	17 (3)	12 (5)	17 (3)	12 (6)
**Serotype**	DENV-1	N/A	133 (17)	N/A	111 (19)	22 (11)	94 (18)	39 (15)	109 (18)	24 (12)
	DENV-2	N/A	153 (19)	N/A	81 (14)	72 (36)	93 (17)	60 (23)	104 (18)	49 (24)
	DENV-3	N/A	403 (51)	N/A	313 (53)	90 (46)	266 (50)	137 (53)	287 (49)	116 (58)
	DENV-3/DENV-4	N/A	1 (<1)	N/A	1 (<1)	0 (0)	0 (0)	1 (1)	0 (0)	1 (<1)
	Indeterminate	N/A	99 (13)	N/A	86 (14)	13 (7)	79 (15)	20 (8)	88 (15)	11 (6)
**Median day of illness (range) at presentation**		4 (1–8)	4 (1–8)	4 (1–7)	4 (1–8)	5 (1–7)	4 (1–8)	4 (1–8)	4 (2–8)	4 (1–8)
**Hospitalization status**	Outpatient	228 (18)	109 (14)	119 (27)	107 (18)	2 (1)	109 (20)	0 (0)	109 (19)	0 (0)
	Inpatient	1,008 (82)	680 (86)	328 (73)	485 (82)	195 (99)	423 (80)	257 (100)	479 (81)	607 (100)
**Median days (range) of hospitalization**		3 (1–6)	3 (1–6)	3 (1–6)	3 (1–5)	3 (1–6)	3 (1–6)	3 (1–6)	3 (1–6)	3 (1–6)

Abbreviations: DENV, dengue virus; WHO, World Health Organization; OFI, other febrile illness; DF, dengue fever; DHF/DSS, dengue hemorrhagic fever/dengue shock syndrome; DWS, dengue with or without warning signs; SD, severe dengue; IC, standardized intervention categories.

**Fig 2:**
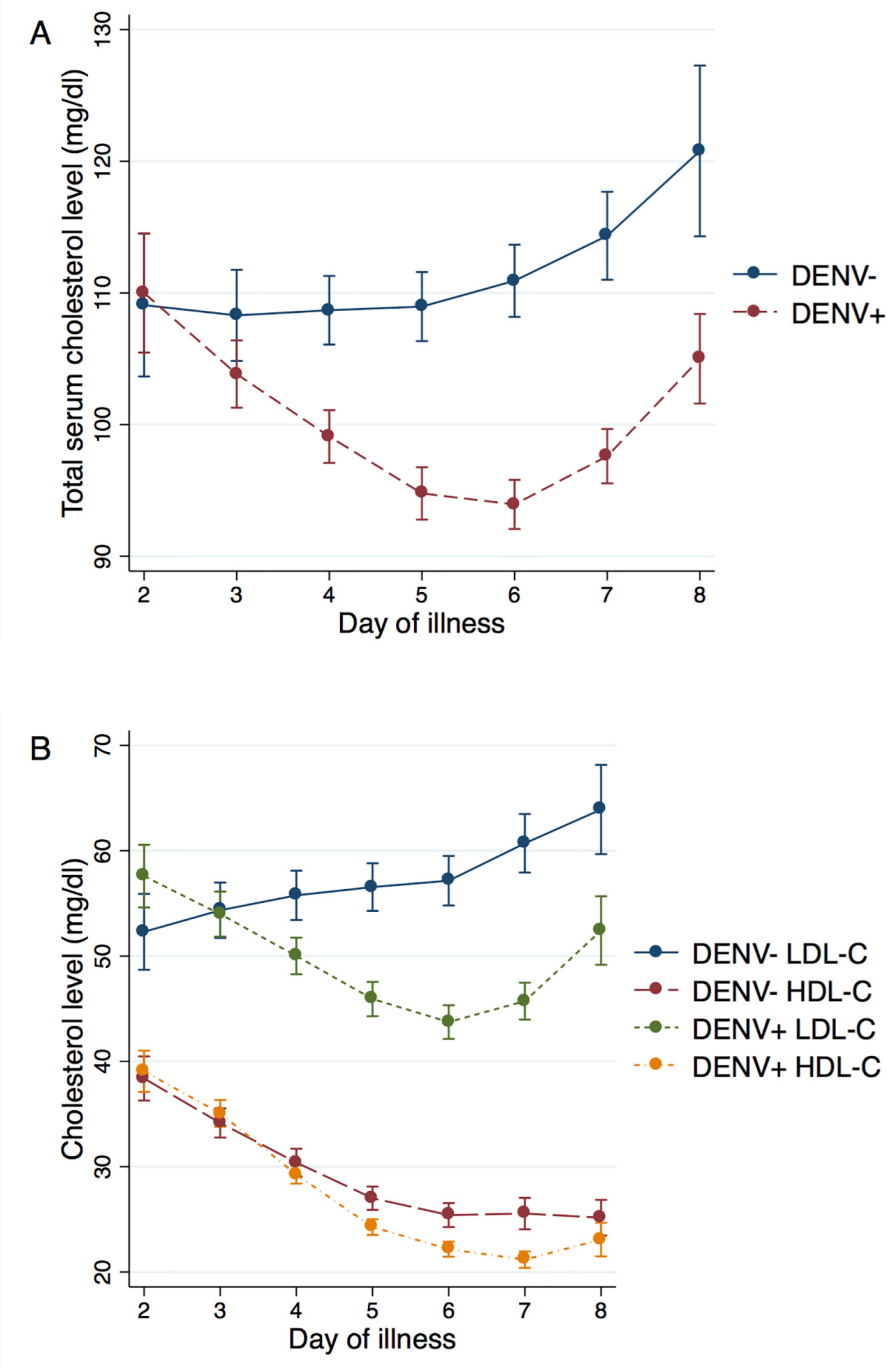
Age- and gender-adjusted marginal mean cholesterol levels (mg/dl) by DENV infection status and day of illness. The day of fever onset was defined as day 1 of illness. **A**, Total serum cholesterol levels were significantly lower in dengue-positive patients compared to dengue-negative patients on days 3–8 of illness (p<0.05). Among dengue-positive patients, LDL-C levels decreased from day 2–6 of illness, and then increased from day 6–8 of illness. However, among dengue-negative patients, total serum cholesterol levels gradually increased from day 2–8 of illness. **B**, Trajectories of LDL-C levels were similar to those of total serum cholesterol levels. Among dengue-positive patients, LDL-C levels decreased from day 2–6 of illness, and then increased from day 6–8 of illness. Among dengue-negative patients, LDL-C levels gradually increased from day 2–8 of illness. LDL-C levels were significantly lower in dengue-positive patients compared to dengue-negative patients on day 2 (p<0.05) and days 4–8 of illness (p<0.001). In contrast, HDL-C levels were significantly lower only on days 5–7 of illness (p<0.001). HDL-C levels followed a similar trajectory among both dengue-positive and dengue-negative patients, decreasing from day 2 to day 6–7 of illness before stabilizing or increasing slightly on day 7–8 of illness. For all cholesterol trajectories, a global test for multiple comparisons across all time-points showed that the differences remained significant at the p<0.05 level.

We delineated the trajectories of cholesterol levels by DENV infection status (Fig 2) and found that total serum cholesterol levels were significantly lower in dengue-positive patients compared to dengue-negative patients on days 3–8 of illness (p<0.05). Among dengue-positive patients, total serum cholesterol levels decreased from day 2 to 6 of illness, and then increased from day 6 to 8 of illness. However, among dengue-negative patients, total serum cholesterol levels gradually increased from day 2 to 8 of illness.

Trajectories of LDL-C levels were similar to those of total serum cholesterol levels. LDL-C levels were significantly lower in dengue-positive patients compared to dengue-negative patients on day 2 (p<0.05) and days 4–8 of illness (p<0.001). In contrast, HDL-C levels were significantly lower only on days 5–7 of illness (p<0.001). HDL-C levels followed a similar trajectory among both dengue-positive and dengue-negative patients, decreasing from day 2 to day 6–7 of illness before stabilizing on day 7–8 of illness. For all cholesterol trajectories, a global test for multiple comparisons across all time-points showed that the differences remained significant at the p<0.05 level.

**Fig 3:**
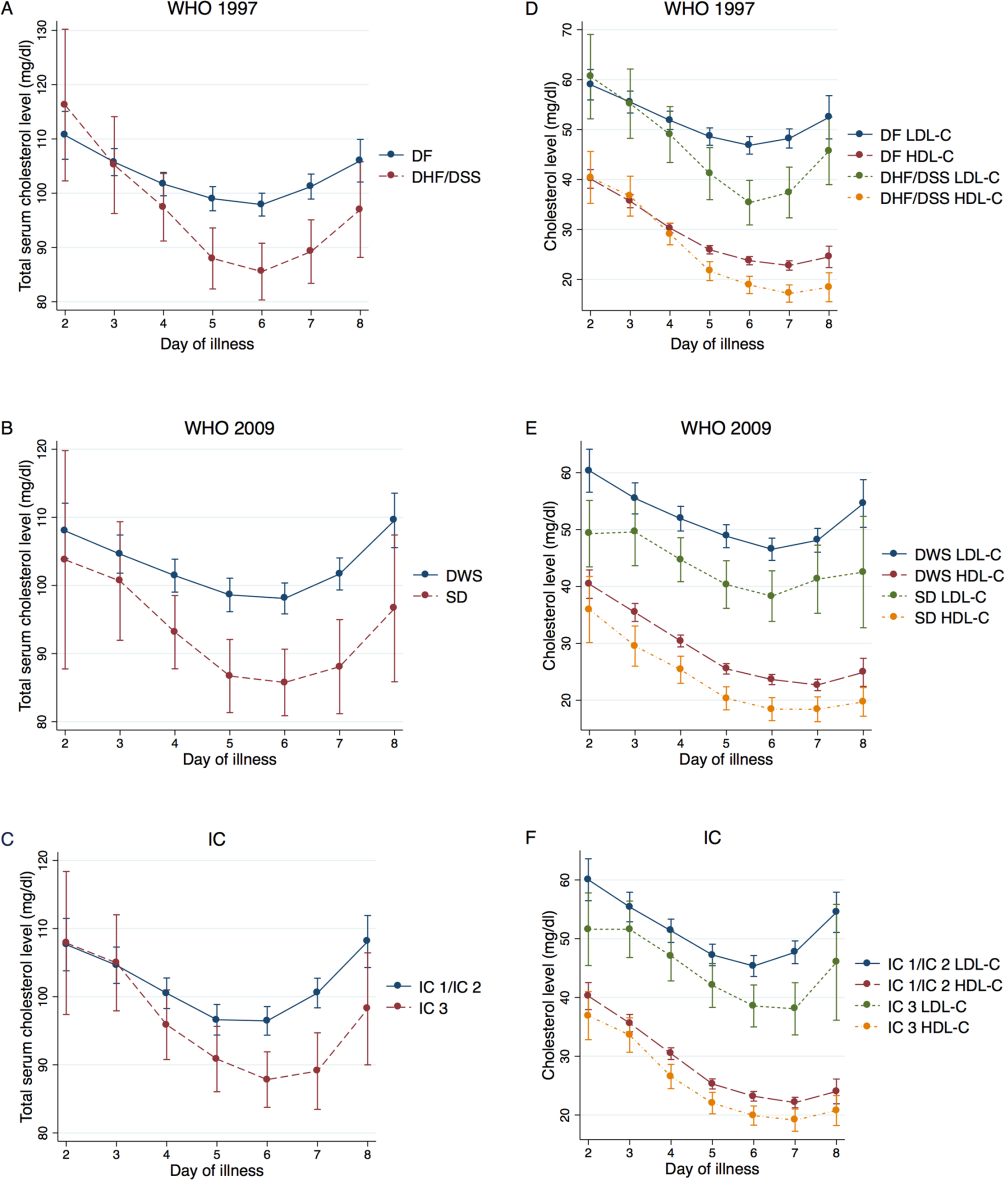
Age- and gender-adjusted marginal mean cholesterol levels (mg/dl) by dengue severity classification and day of illness. For the WHO 1997 classification, mild dengue was defined as DF and severe dengue was defined as DHF/DSS. For the WHO 2009 classification, mild dengue was defined as Dengue with and without Warning Signs and severe dengue was defined as SD. For the standardized intervention categories, mild dengue was defined as IC 1/IC 2 care and severe dengue was defined as IC 3 care. The day of fever onset was defined as day 1 of illness. **A**, **B**, **C**, Total serum cholesterol levels were significantly lower in patients who developed severe dengue compared to patients with mild dengue on days 5–7 of illness using the WHO 1997 classification (p<0.001), on days 4–8 of illness using the WHO 2009 classification (p<0.05), and on days 5–8 of illness using standardized intervention categories (p<0.05). Regardless of dengue outcome, total serum cholesterol levels decreased from day 2–6 and increased from day 6–8 of illness. **D**, **E**, **F**, LDL-C levels were significantly lower in patients who developed severe dengue compared to patients with mild dengue on days 5–7 of illness using the WHO 1997 classification (p<0.01), days 4–8 of illness using the WHO 2009 classification (p<0.05), and on day 2 and days 5–7 of illness using standardized intervention categories (p<0.05). Regardless of dengue outcome, LDL-C levels decreased from day 3–6 and increased from day 6–8 of illness. HDL-C levels were significantly lower in patients who developed severe dengue compared to patients with mild dengue on days 5–8 of illness using the WHO 1997 classification (p<0.01), days 3–8 of illness using the WHO 2009 classification (p<0.01), and days 4–8 of illness using standardized intervention categories (p≤0.05). Regardless of dengue outcome, HDL-C levels decreased from day 2–7 of illness before increasing slightly on day 8 of illness.

We also examined the trajectories of cholesterol by dengue severity (Fig 3). Here the analysis was restricted to patients who were classified as mild dengue at presentation and, if they developed severe dengue, developed severe dengue >12 hours after presentation. Total serum cholesterol levels were significantly lower in patients who developed severe dengue compared to patients with mild dengue on days 5–7 of illness using the WHO 1997 classification (p<0.001), on days 4–8 of illness using the WHO 2009 classification (p<0.05), and on days 5–8 of illness using standardized intervention categories (p<0.05). LDL-C levels were significantly lower in patients who developed severe dengue compared to patients with mild dengue on days 5–7 of illness using the WHO 1997 classification (p<0.01), days 4–8 of illness using the WHO 2009 classification (p<0.05), and on day 2 and days 5–7 of illness using standardized intervention categories (p<0.05). HDL-C levels were significantly lower in patients who developed severe dengue disease compared to patients with mild dengue on days 5–8 of illness using the WHO 1997 classification (p<0.01), days 3–8 of illness using the WHO 2009 classification (p<0.01), and days 4–8 of illness using standardized intervention categories (p≤0.05). Overall, regardless of dengue outcome, both total serum cholesterol and LDL-C levels decreased from day 3–6 and increased from day 6–8 of illness. Similarly, HDL-C levels decreased from day 2–7 of illness before increasing slightly on day 8 of illness.

Furthermore, we examined the association of cholesterol levels with albumin levels (a marker of vascular leakage), adjusting for DENV infection status and day of illness. Total serum cholesterol, LDL-C and HDL-C levels were each positively correlated with albumin levels (p<0.0001 for each comparison). As shown in S3A Fig, median total serum cholesterol levels followed a similar pattern to median albumin levels, decreasing over the course of illness. Overall, dengue-positive patients had lower median total cholesterol and albumin levels than patients with OFI on all days of illness. In addition, median total cholesterol and albumin levels were lower in severe dengue cases than in mild dengue cases over the course of illness (S3B–S3D Fig). This was observed in all disease severity classification schemes, with the steepest decline apparent in DHF/DSS cases on days 4–6 (S3B Fig).

Finally, we constructed multivariable models to examine the effect of cholesterol level at presentation on subsequent risk of development of severe dengue as defined by the three classification schemes. Using the WHO 1997 disease severity classification, we found that for each 10 mg/dl decrease in total serum cholesterol and LDL-C at presentation, risk of development of DHF/DSS increased by 9% (95% CI: 0–19%) and 12% (95% CI: 0–26%), respectively (Table 2). A 10 mg/dl decrease in HDL-C at presentation was not significantly associated with risk of development of DHF/DSS. We also examined the effect of total serum cholesterol, LDL-C and HDL-C at presentation on risk of development of severe dengue as defined by the WHO 2009 classification and standardized intervention categories, but none of the findings were statistically significant.

**Table 2:** Effect of cholesterol level at presentation on development of severe dengue outcome using the WHO 1997 disease severity classification.*

	Cholesterol type		
	Total serum cholesterol	LDL-C	HDL-C
Variable	RR (95% CI)	RR (95% CI)	RR (95% CI)
Cholesterol per -10 mg/dl	1.09 (1.00–1.19)	1.12 (1.00–1.26)	1.18 (0.98–1.42)
Age (years)	1.06 (1.00–1.14)	1.06 (0.99–1.14)	1.06 (0.99–1.13)
Female	1.04 (0.69–1.56)	0.99 (0.64–1.51)	0.96 (0.63–1.45)
Malnutrition	0.76 (0.33–1.75)	0.73 (0.29–1.80)	0.78 (0.34–1.78)
Secondary immune response	1.98 (1.22–3.19)	1.91 (1.17–3.14)	1.85 (1.15–2.98)

*Severe dengue outcome was modeled by cholesterol at presentation, age, gender, malnutrition and immune response using modified Poisson models with robust standard errors. For the WHO 1997 disease severity classification, severe dengue outcome was defined as DHF/DSS and mild dengue outcome, the reference group, was defined as DF.

Abbreviations: WHO, World Health Organization; LDL-C, low-density lipoprotein cholesterol; HDL-C, high-density lipoprotein cholesterol; DHF/DSS, dengue hemorrhagic fever/dengue shock syndrome; DF, dengue fever.

## Discussion

While other studies have examined cholesterol levels during a particular phase of dengue illness (acute, critical or convalescent) [19,20] or on day of admission to the hospital [17,18], ours is the first study of which we are aware to analyze changes in cholesterol levels by day of illness in dengue patients, and to use time-ordered analysis that enables prediction of disease severity level based on cholesterol levels at presentation. We found that total serum cholesterol, LDL-C, and HDL-C levels were significantly lower in dengue-positive patients compared to dengue-negative patients, and that LDL-C levels showed greater decreases and thus appeared to drive the reduction in total cholesterol. Total, LDL-C, and HDL-C levels were lower in severe compared to mild dengue during the course of illness regardless of severity classification scheme. Finally, we found that lower total serum cholesterol and LDL-C levels at presentation were associated with subsequent development of DHF/DSS.

Liver damage caused by DENV infection could be contributing to the lower cholesterol levels we observed in dengue patients. Liver damage is a well-established characteristic of dengue patients [21], particularly severe cases, and higher liver enzyme levels (aspartate aminotransferase and alanine aminotransferase) have been associated with increasing dengue severity across different classification schemes [43]. The liver is a major site of cholesterol synthesis in humans, and the rate of cholesterol production depends on the cellular level of cholesterol, for which LDL and HDL, among other lipoproteins, are responsible through their roles in cholesterol transport [44].

When we further examined lower levels of LDL-C and HDL-C during DENV infection, we found that although HDL-C and LDL-C both decreased over the course of dengue illness, there were greater decreases in LDL-C among dengue-positive patients compared to patients with OFI, suggesting that the reduction in LDL-C is likely driving the decrease in total serum cholesterol. This finding is supported by that of Seet and colleagues, who found greater decreases in LDL-C compared to HDL-C among DF cases during the acute and critical phases of dengue compared to levels during convalescence [20]. *In vitro* studies have shown that the related hepatitis C virus can enter cells via LDL receptors [7], and increased expression of LDL receptors in Huh-7 cells has been reported to alter DENV infection, triggering increased uptake of LDL particles in infected compared to non-infected cells [15]. Our finding of lower HDL-C levels in severe dengue cases compared to mild dengue cases is intriguing. An *in vitro* study by Li and colleagues has shown that ApoAI, a major HDL apolipoprotein, binds to DENV and is associated with enhanced virus infection [45]. Therefore, the decrease in HDL-C observed among severe cases may be due to lack of available ApoAI, possibly in addition to reduced ApoAI production due to liver dysfunction.

We also found that lower total serum cholesterol and LDL-C levels at presentation were associated with subsequent risk of developing severe dengue using the WHO 1997 dengue severity classification. Suvarna and colleagues similarly showed that LDL-C levels were associated with higher odds of DHF [18]. However, while previous studies have shown differences in total serum cholesterol levels by dengue severity using basic statistical tests, they did not find statistically significant associations between lower total cholesterol and severe dengue using multivariable models [19]. A major strength of our study is that we had prospective follow-up of dengue patients over the course of their illness, which allowed us to account for time ordering of the exposure (cholesterol) before the outcome (severe dengue). By restricting our analyses to patients who were classified as mild dengue at presentation and, if they developed severe dengue, developed severe dengue >12 hours after presentation, we were able to assess the effect of cholesterol at presentation on development of severe dengue without confounding it with the effect of severe dengue on cholesterol. The ability to account for time ordering is likely the reason for the differences in our results compared to those of other studies.

In our study, we analyzed severe dengue using three different dengue severity classifications. Although the WHO released a revised classification scheme in 2009 [22], it has been somewhat controversial with some arguing that it may result in the misclassification of mild dengue cases as severe or overburden medical centers in dengue-endemic countries [33,46,47]. In one study, Narvaez and colleagues show that while the revised scheme had higher sensitivity and specificity to identify cases in need of intensive clinical intervention, it was less specific to a particular pathophysiology (e.g., vascular leakage leading to shock) than the traditional WHO 1997 classification scheme [24]. The primary difference between the two classification schemes is the centrality of the vascular leakage syndrome in the WHO 1997 classification, whereas the 2009 WHO classification includes other pathophysiological mechanisms associated with severe dengue disease. In our study, using the WHO 1997 classification, total serum cholesterol and LDL-C were associated with risk of development of severe dengue, whereas HDL-C was not. Interestingly, using the WHO 2009 classification and standardized intervention categories, none of the cholesterol types were associated with risk of development of severe dengue. These results suggest that the association of cholesterol with severe dengue outcome is specific to the pathophysiology of DHF/DSS and not just the pathophysiology of severe illness. Although previous studies have associated low cholesterol with critical illness [48], meningococcal sepsis [49], and more hospital admissions for infectious disease [50], it is possible that not accounting for time ordering or confounders may explain these associations or that other infectious diseases may share similar pathogenic pathways.

The increased endothelial permeability that is associated with severe dengue disease [51] could potentially enable leakage of cholesterol molecules, resulting in lower cholesterol levels measured within the circulatory system [52]. Our results showing daily cholesterol levels decreasing alongside daily albumin levels in dengue-positive patients, especially among patients with severe dengue, supports this idea. However, LDL-C has a substantially larger molecular weight than albumin [53]. Nonetheless, studies have shown that cholesterol in the form of LDL can be transported across the microvascular endothelial barrier [54], and in the form of oxidized LDL can promote vascular leakage [55,56]. Other potential explanations for the decrease of serum cholesterol in dengue, and in particular severe dengue, include accumulation in the liver where hepatic steatosis is observed [57], uptake by monocyte-derived macrophages [58], or uptake by DENV-infected cells, as cholesterol is involved in flavivirus entry and replication [4–6,8,9]. More research is needed to specifically investigate the mechanisms that account for the decreased levels of circulating cholesterol and the association of lower cholesterol levels with severe dengue outcome.

A large number of severe dengue cases was available for analysis due to the patient population of children, who bear the burden of DHF/DSS in Nicaragua; furthermore, the study was based at the National Pediatric Reference Hospital, which treats the majority of pediatric dengue cases in Nicaragua. Interestingly, a substantial proportion of the DHF/DSS cases were in primary infections; this occurred because a large number of dengue cases were due to DENV-3 since it was the dominant serotype circulating during several years of the study, and DENV-3 is known to cause more DHF/DSS in primary infections than other serotypes (particularly compared to DENV-2 and DENV-4) [28,59–61].

Our study had several methodological strengths. We used a directed acyclic graph to guide the construction of our statistical models to ensure that we only adjusted for plausible confounders, thereby avoiding bias. Although our directed acyclic graph is considered a working diagram and therefore could be modified in the future, it is a transparent approach to model-building that relies on our current knowledge rather than the statistical significance of covariates, which may, in fact, reflect relationships with parameters other than the outcome. We also used statistical methods that enabled calculation of the cumulative incidence ratio (relative risk) rather than the odds ratio, which may overestimate the relationship between cholesterol and dengue outcome when disease is common [62]. In addition, our study design compared the trajectories of cholesterol levels in dengue cases to those in patients with OFI rather than to those in healthy controls. Patients with OFI, not healthy individuals, are the individuals who present as suspected dengue cases to clinics and hospitals and therefore are the more relevant comparison group for dengue cases. Finally, we accounted for time-ordering in our analyses, which ensured that the exposure preceded the outcome.

Our study did have some limitations. It would have been interesting to examine changes in cholesterol levels from baseline values in individuals with dengue over the course of illness. While pre-infection levels would be very difficult to obtain, convalescent samples could have provided a reasonable estimate of baseline values. Unfortunately, cholesterol measurements were not routinely performed on these samples. Prospective studies would enable capture of baseline cholesterol values, with cholesterol measured at convalescence as well. LDL-C was not measured in the first two of the nine years of the hospital study, so we had somewhat fewer measurements available for analysis, and triglycerides were not measured. As the third component of total serum cholesterol in addition to HDL-C and LDL-C, triglyceride levels would have helped us to understand whether LDL-C alone was driving the reduction in total cholesterol levels or whether triglycerides also contributed. In one study, triglycerides <150 mg/dl were estimated to increase the odds of DSS by 41%, although this association was not significant [18].

Although of potential concern, the fact that we obtained non-fasting cholesterol measurements should not have affected our findings. Recent studies have found the impact of eating on cholesterol measurements to be very limited [63,64]. According to Langsted and colleagues, “Lipid profiles at most change minimally in response to normal food intake in individuals in the general population” [64]. In their study, the maximum changes in lipid profiles after normal food and fluid intake from fasting levels were 0.2 mmol/L for total cholesterol, 0.2 mmol/L for low-density lipoprotein cholesterol, and 0.1 mmol/L for HDL cholesterol [64]. In addition, eating should not influence the relationship between cholesterol and severe dengue outcome because eating was not a confounder in our directed acyclic graph (see S1 Fig). Nutritional status, however, was considered a confounder, as some studies have shown less severe dengue among malnourished children [65–67], so it was included in our graph.

In summary, our results show that lower total serum cholesterol and LDL-C levels at presentation were associated with subsequent risk of developing severe dengue using the WHO 1997 dengue severity classification and suggest that the reduction in LDL-C is likely driving the decreases observed in total serum cholesterol levels among dengue-positive patients. In addition, they indicate that cholesterol level at presentation may serve as a potential predictor of severe dengue. The burden of dengue is expected to continue to increase in the future due to climate change, globalization, travel, trade, urbanization, socioeconomics, viral evolution and other factors [68]. Therefore, time is of the essence for developing better diagnostic and prognostic tools to identify severe dengue cases for the provision of appropriate supportive care and hopefully, one day, specific therapeutics. Cholesterol and other routine laboratory markers should be explored as a lower cost and more sustainable approach to developing biomarker panels as prognostic markers of severe dengue.

## Supporting Information

S1 Fig Directed acyclic graph of the association between cholesterol at presentation and severe dengue outcome >12 hours after presentation. Solid lines indicate established associations and dashed lines indicate possible associations. Immune response refers to secondary versus primary immune response. Nutrition refers to nutritional status established over time, whereas eating refers to temporal nutrition.
(TIF)

S2 Fig Median cholesterol levels by day of illness and disease severity classification. *A*, *B*, *C*. Median cholesterol values were calculated separately for each disease severity group by day of illness. Regardless of disease severity classification, median total serum cholesterol levels generally decreased over the course of illness until day 5 or 6 of illness. In addition, more severe cases tended to have lower median total serum cholesterol levels than less severe cases and patients with OFI. *D*, *E*, *F*. Median LDL-C levels followed a similar pattern to median total serum cholesterol levels. *G*, *H*, *I*. Median HDL-C levels generally decreased over the course of illness until day 6 or 7 of illness. More severe cases tended to have lower median HDL-C levels than less severe cases and patients with OFI. Data for DSS cases on day 3 of illness are not shown due to low counts. Abbreviations: OFI, other febrile illness; DF, dengue fever; DHF, dengue hemorrhagic fever; DSS, dengue shock syndrome; DwoWS, dengue without warning signs; DwWS, dengue with warning signs; SD, severe dengue; IC 1–3, intervention categories 1–3; LDL-C, low-density lipoprotein cholesterol; HDL-C, high-density lipoprotein cholesterol.
(TIF)

S3 Fig Median total serum cholesterol levels and albumin levels by DENV infection status, dengue severity classification and day of illness. *A*, *B*, *C*, *D*. Median total serum cholesterol levels and median albumin levels were calculated separately by DENV infection status and by dengue severity classification for each day of illness. Median total serum cholesterol levels followed a similar pattern to median albumin levels, decreasing over the course of illness. Dengue-positive patients had lower median cholesterol and albumin levels than patients with OFI on all days of illness. In addition, median total cholesterol and albumin levels tended to be lower in severe dengue cases than in mild dengue cases on all days of illness, regardless of severity classification scheme. Abbreviations: OFI, other febrile illness; TC, total serum cholesterol; DENV, dengue virus; ALB, albumin; DF, dengue fever; DHF, dengue hemorrhagic fever; DSS, dengue shock syndrome; DWS, dengue with or without warning signs; SD, severe dengue; IC 1–3, intervention categories 1–3.
(TIF)

S1 Table Computerized algorithm to classify laboratory-confirmed dengue cases according to the 1997 and 2009 WHO classifications of dengue disease severity. (XLSX)

S2 Table Classifications of dengue disease severity used in this study. (DOCX)

S3 Table Patient counts by day of illness and WHO classification criteria. (DOCX)

S4 Table STROBE checklist for cohort studies. (DOCX)
