# Supplementary figures and images for: Lower Low-Density Lipoprotein Cholesterol Levels Are Associated with Severe Dengue Outcome
Source: PLoS Negl Trop Dis. 2015 Sep 3;9(9):e0003904. doi: 10.1371/journal.pntd.0003904 (PMC4559460; doi:10.1371/journal.pntd.0003904)

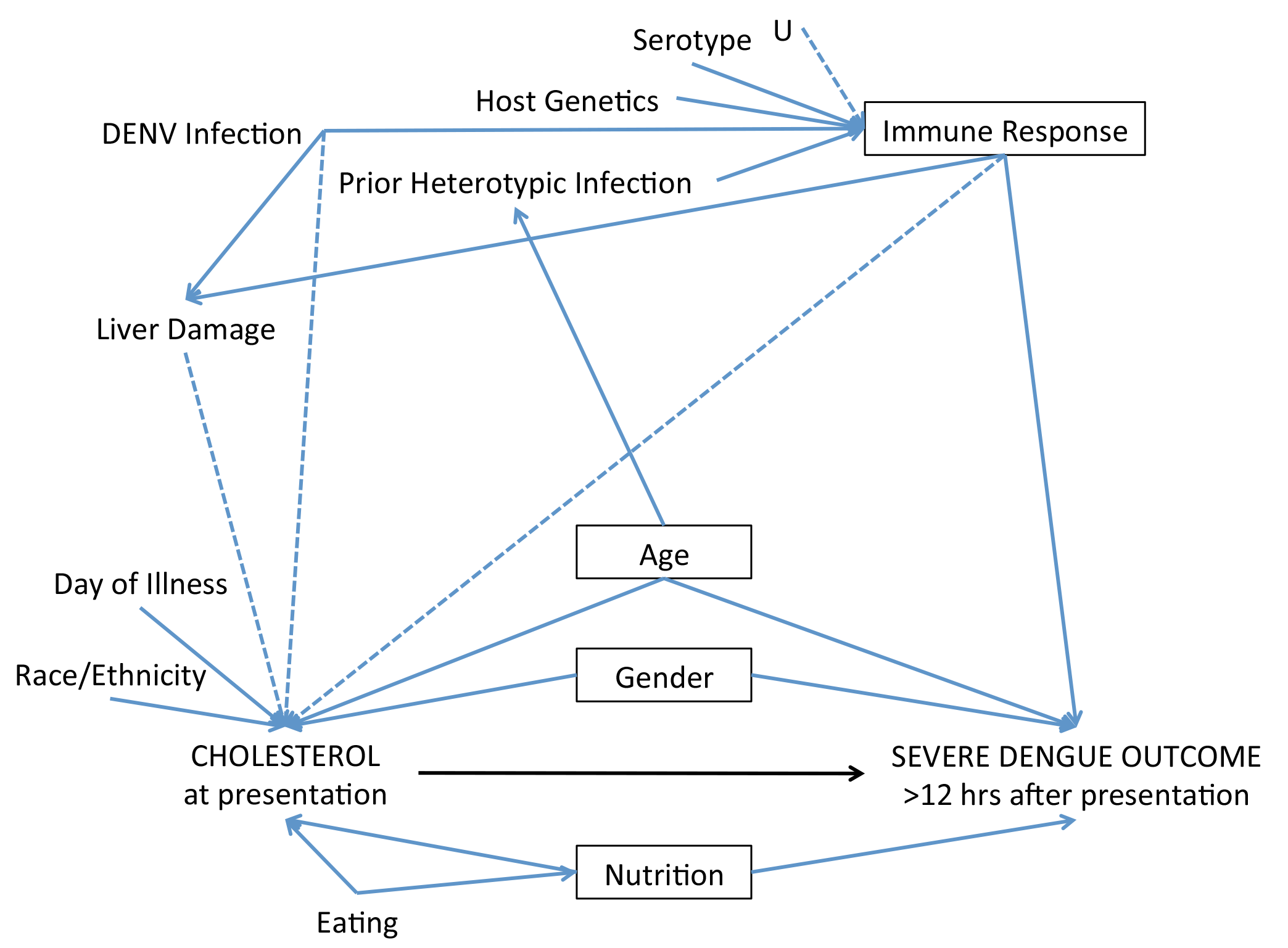

Supplement: S1 Fig — Solid lines indicate established associations and dashed lines indicate possible associations. Immune response refers to secondary versus primary immune response. Nutrition refers to nutritional status established over time, whereas eating refers to temporal nutrition. (TIF) [file pntd.0003904.s001.tif]

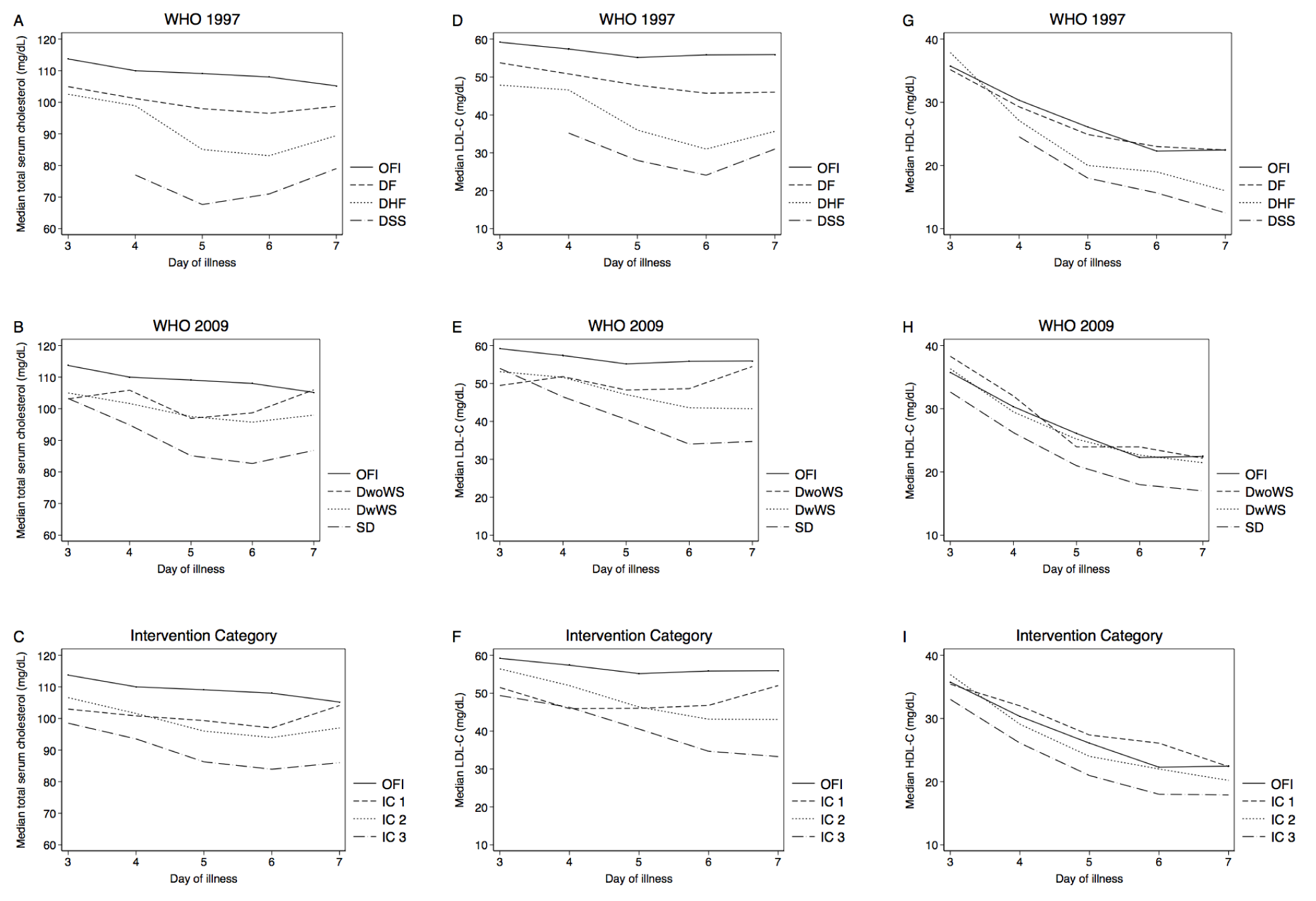

Supplement: S2 Fig — A, B, C. Median cholesterol values were calculated separately for each disease severity group by day of illness. Regardless of disease severity classification, median total serum cholesterol levels generally decreased over the course of illness until day 5 or 6 of illness. In addition, more severe cases tended to have lower median total serum cholesterol levels than less severe cases and patients with OFI. D, E, F. Median LDL-C levels followed a similar pattern to median total serum cholesterol levels. G, H, I. Median HDL-C levels generally decreased over the course of illness until day 6 or 7 of illness. More severe cases tended to have lower median HDL-C levels than less severe cases and patients with OFI. Data for DSS cases on day 3 of illness are not shown due to low counts. Abbreviations: OFI, other febrile illness; DF, dengue fever; DHF, dengue hemorrhagic fever; DSS, dengue shock syndrome; DwoWS, dengue without warning signs; DwWS, dengue with warning signs; SD, severe dengue; IC 1–3, intervention categories 1–3; LDL-C, low-density lipoprotein cholesterol; HDL-C, high-density lipoprotein cholesterol. (TIF) [file pntd.0003904.s002.tif]

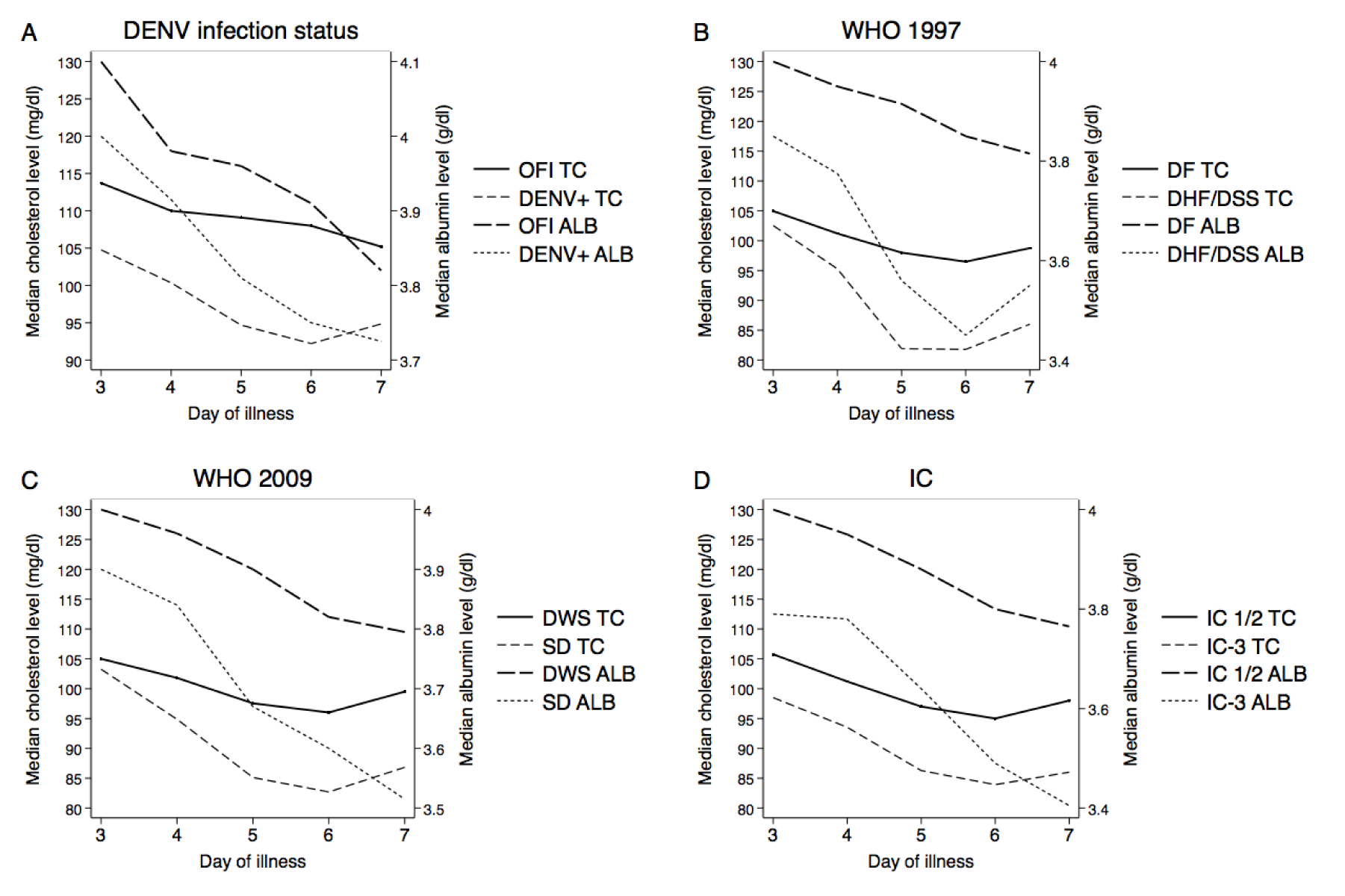

Supplement: S3 Fig — A, B, C, D. Median total serum cholesterol levels and median albumin levels were calculated separately by DENV infection status and by dengue severity classification for each day of illness. Median total serum cholesterol levels followed a similar pattern to median albumin levels, decreasing over the course of illness. Dengue-positive patients had lower median cholesterol and albumin levels than patients with OFI on all days of illness. In addition, median total cholesterol and albumin levels tended to be lower in severe dengue cases than in mild dengue cases on all days of illness, regardless of severity classification scheme. Abbreviations: OFI, other febrile illness; TC, total serum cholesterol; DENV, dengue virus; ALB, albumin; DF, dengue fever; DHF, dengue hemorrhagic fever; DSS, dengue shock syndrome; DWS, dengue with or without warning signs; SD, severe dengue; IC 1–3, intervention categories 1–3. (TIF) [file pntd.0003904.s003.tif]
