# Supplementary material for: Lower Low-Density Lipoprotein Cholesterol Levels Are Associated with Severe Dengue Outcome
Source: PLoS Negl Trop Dis. 2015 Sep 3;9(9):e0003904. doi: 10.1371/journal.pntd.0003904 (PMC4559460; doi:10.1371/journal.pntd.0003904)
Supplement: S2 Table — (DOCX) [file pntd.0003904.s005.docx]

**Table S2. Classifications of dengue disease severity.***

| **WHO 1997 Criteria** | **WHO 2009 Criteria** | **Intervention Category** |
| --- | --- | --- |
| Dengue Fever | Dengue without Warning Signs | Category 1 (Standard) |
| Acute febrile illness with two or more of the following:   - Headache - Retro-orbital pain - Myalgia - Leukopenia - Arthralgia - Rash - Hemorrhagic manifestations | Fever and two of the following:   - Nausea, vomiting - Rash - Aches and pains - Leukopenia - Positive tourniquet test | - Patients who were managed as outpatients and did not present criteria for hospitalization |
| Dengue Hemorrhagic Fever (DHF) | Dengue with Warning Signs | Category 2 (Intermediate) |
| All of the following must be present:   - Fever or history of acute fever lasting 2–7 days - Hemorrhagic manifestations: - positive tourniquet test; - petechiae, equimosis, purpura or bleeding from mucosa, gastrointestinal tract, injection sites or other locations; - hematemesis; - melena - Thrombocytopenia (≤100,000 platelets/mm^3^) - Evidence of plasma leakage due to increased vascular permeability | Dengue as defined above with any of the following:   - Abdominal pain or tenderness - Persistent vomiting - Clinical fluid accumulation - Mucosal bleeding - Lethargy, restlessness - Liver enlargement >2 cm - Laboratory: increase in HCT concurrent with rapid decrease in platelet count | - Hospitalized patients who received intravenous fluids for rehydration or maintenance and did not suffer organ damage |
| Dengue Shock Syndrome (DSS) | Severe Dengue | Category 3 (Major) |
| DHF with hypotension for age or narrow pulse pressure (<=20 mmHg) plus one of the following:   - rapid and weak pulse; - cold, clammy skin; - restlessness; - poor capillary refill (>2 sec) | Dengue with at least one of the following criteria:   - Severe plasma leakage leading to: - Shock (DSS) - Fluid accumulation with respiratory distress - Severe bleeding as evaluated by clinician - Severe organ involvement - Liver: AST or ALT≥1000 IU - CNS: impaired consciousness - Failure of heart and other organs | - Patients hospitalized in the Intensive Care Unit, administered inotropic drugs or ventilation, or who experienced organ failure |

*In this study, severe and mild dengue were defined as follows: for the WHO 1997 classification, mild dengue was defined as DF and severe dengue was defined as DHF or DSS. For the WHO 2009 classification, mild dengue was defined as dengue with or without warning signs and severe dengue was defined verbatim. For the standardized intervention categories, mild dengue was defined as intervention category (IC) 1/IC 2 care and severe dengue was defined as IC 3 care.
