# Supplementary material for: Lower Low-Density Lipoprotein Cholesterol Levels Are Associated with Severe Dengue Outcome
Source: PLoS Negl Trop Dis. 2015 Sep 3;9(9):e0003904. doi: 10.1371/journal.pntd.0003904 (PMC4559460; doi:10.1371/journal.pntd.0003904)
Supplement: S3 Table — (DOCX) [file pntd.0003904.s006.docx]

**Table S3**. Patient counts by day of illness and WHO classification criteria.*

|  |  |  |  |  | WHO 1997 | | WHO 2009 | |  |
| --- | --- | --- | --- | --- | --- | --- | --- | --- | --- |
| Day of illness |  | Total  (n=3,441) | DENV+  (n=2,222) | OFI  (n=1,219) | DF  (n=1,631) | DHF/DSS  (n=591) | DWS  (n=1,485) | SD  (n=737) | |
|  |  | n (%) | n (%) | n (%) | n (%) | n (%) | n (%) | n (%) | |
| 2 |  | 141 (4) | 72 (3) | 69 (6) | 60 (4) | 12 (2) | 34 (2) | 38 (5) | |
| 3 |  | 390 (11) | 214 (10) | 176 (15) | 178 (11) | 36 (6) | 137 (9) | 77 (10) | |
| 4 |  | 667 (19) | 408 (18) | 259 (21) | 327 (20) | 81 (14) | 276 (19) | 132 (18) | |
| 5 |  | 889 (26) | 587 (26) | 302 (25) | 434 (27) | 153 (26) | 401 (27) | 186 (25) | |
| 6 |  | 762 (22) | 526 (24) | 236 (19) | 375 (23) | 151 (25) | 365 (25) | 161 (22) | |
| 7 |  | 435 (13) | 312 (14) | 123 (10) | 202 (12) | 110 (19) | 211 (14) | 101 (14) | |
| 8 |  | 157 (5) | 103 (5) | 54 (4) | 55 (3) | 48 (8) | 61 (4) | 42 (6) | |

*Patients contributed data on multiple days of illness; therefore, the counts listed are greater than the unique number of patients in each group.

Abbreviations: DENV, dengue virus infection; WHO, World Health Organization; DENV+, laboratory-confirmed positive for dengue virus infection; OFI, other febrile illness; DF, dengue fever; DHF/DSS, dengue hemorrhagic fever/dengue shock syndrome; DWS, dengue with or without warning signs; SD, severe dengue.
