# Supplementary material for: Lower Low-Density Lipoprotein Cholesterol Levels Are Associated with Severe Dengue Outcome
Source: PLoS Negl Trop Dis. 2015 Sep 3;9(9):e0003904. doi: 10.1371/journal.pntd.0003904 (PMC4559460; doi:10.1371/journal.pntd.0003904)
Supplement: S4 Table — (DOCX) [file pntd.0003904.s007.docx]

**Table S2. STROBE checklist for cohort studies.**

|  | Item No | Recommendation |
| --- | --- | --- |
| **Title and abstract** | 1 | (*a*) Indicate the study’s design with a commonly used term in the title or the abstract  **Done - see Abstract** |
|  |  | (*b*) Provide in the abstract an informative and balanced summary of what was done and what was found  **Done** |
| Introduction | | |
| Background/rationale | 2 | Explain the scientific background and rationale for the investigation being reported  **Done** |
| Objectives | 3 | State specific objectives, including any prespecified hypotheses  **Done** |
| Methods | | |
| Study design | 4 | Present key elements of study design early in the paper  **Done – see Study site and population subsection** |
| Setting | 5 | Describe the setting, locations, and relevant dates, including periods of recruitment, exposure, follow-up, and data collection  **Done – see Study site and population and Data collection subsections** |
| Participants | 6 | (*a*) Give the eligibility criteria, and the sources and methods of selection of participants. Describe methods of follow-up  **Done – see Study site and population and Data collection subsections** |
|  |  | (*b*) For matched studies, give matching criteria and number of exposed and unexposed  **N/A** |
| Variables | 7 | Clearly define all outcomes, exposures, predictors, potential confounders, and effect modifiers. Give diagnostic criteria, if applicable  **Done – see Cholesterol measurements, Dengue diagnosis, Dengue disease outcome and Statistical analysis subsections, also Figure S1** |
| Data sources/ measurement | 8* | For each variable of interest, give sources of data and details of methods of assessment (measurement). Describe comparability of assessment methods if there is more than one group  **Done – see Cholesterol measurements, Dengue diagnosis, Dengue disease outcome and Statistical analysis subsections, also Table S1** |
| Bias | 9 | Describe any efforts to address potential sources of bias  **Done – see Statistical analysis subsection, also Figure S1** |
| Study size | 10 | Explain how the study size was arrived at  **Done – see Study site and population subsection, also Figure 1** |
| Quantitative variables | 11 | Explain how quantitative variables were handled in the analyses. If applicable, describe which groupings were chosen and why  **Done – see Dengue disease outcome and Statistical analysis subsections, also Table S1** |
| Statistical methods | 12 | (*a*) Describe all statistical methods, including those used to control for confounding  **Done – see Statistical analysis subsection, also Figure S1** |
|  |  | (*b*) Describe any methods used to examine subgroups and interactions  **Done – see Statistical analysis subsection** |
|  |  | (*c*) Explain how missing data were addressed  **Done – see Statistical analysis subsection, also Results** |
|  |  | (*d*) If applicable, explain how loss to follow-up was addressed  **N/A for these analyses** |
|  |  | (*e*) Describe any sensitivity analyses  **N/A** |
| Results | | |
| Participants | 13* | (a) Report numbers of individuals at each stage of study—eg numbers potentially eligible, examined for eligibility, confirmed eligible, included in the study, completing follow-up, and analysed  **Done – see Figure 1** |
|  |  | (b) Give reasons for non-participation at each stage |
|  |  | (c) Consider use of a flow diagram  **Done – see Figure 1** |
| Descriptive data | 14* | (a) Give characteristics of study participants (eg demographic, clinical, social) and information on exposures and potential confounders  **Done – see Table 1** |
|  |  | (b) Indicate number of participants with missing data for each variable of interest  **Done – see Table 1** |
|  |  | (c) Summarise follow-up time (eg, average and total amount)  **Done – see Table 1** |
| Outcome data | 15* | Report numbers of outcome events or summary measures over time  **Done – see Table 1** |
| Main results | 16 | (*a*) Give unadjusted estimates and, if applicable, confounder-adjusted estimates and their precision (eg, 95% confidence interval). Make clear which confounders were adjusted for and why they were included  **Done – see Tables 2 and 3, also Figure S1** |
|  |  | (*b*) Report category boundaries when continuous variables were categorized  **Done** |
|  |  | (*c*) If relevant, consider translating estimates of relative risk into absolute risk for a meaningful time period  **Not relevant** |
| Other analyses | 17 | Report other analyses done—eg analyses of subgroups and interactions, and sensitivity analyses  **Done** |
| Discussion | | |
| Key results | 18 | Summarise key results with reference to study objectives  **Done** |
| Limitations | 19 | Discuss limitations of the study, taking into account sources of potential bias or imprecision. Discuss both direction and magnitude of any potential bias  **Done** |
| Interpretation | 20 | Give a cautious overall interpretation of results considering objectives, limitations, multiplicity of analyses, results from similar studies, and other relevant evidence  **Done** |
| Generalisability | 21 | Discuss the generalisability (external validity) of the study results  **Done** |
| Other information | | |
| Funding | 22 | Give the source of funding and the role of the funders for the present study and, if applicable, for the original study on which the present article is based  **Done** |
